# The Effect of Chronic Intermittent Hypobaric Hypoxia on Sleep Quality and Melatonin Serum Levels in Chilean Miners

**DOI:** 10.3389/fphys.2021.809360

**Published:** 2022-02-09

**Authors:** Rodrigo Calderon-Jofre, Daniel Moraga, Fernando A. Moraga

**Affiliations:** ^1^Laboratorio de Fisiología, Hipoxia y Función Vascular, Departamento de Ciencias Biomédicas, Facultad de Medicina, Universidad Católica del Norte, Coquimbo, Chile; ^2^Departamento de Medicina, Facultad de Ciencias de la Salud, Universidad de Tarapacá, Arica, Chile

**Keywords:** melatonin, chronic intermittent hypobaric hypoxia, sleep quality, oxygen saturation, heart rate

## Abstract

High-altitude mining is an important economic resource for Chile. These workers are exposed to chronic intermittent hypobaric hypoxia (CIHH), which reduces their sleep quality and increases the risk of accidents and long-term illnesses. Melatonin, a hormone produced by the pineal gland, is a sleep inducer that regulates the circadian cycle and may be altered in populations subjected to CIHH. This work aimed to assess the relationship between altitude, sleep quality, and plasma melatonin concentrations in miners with CIHH exposure. 288 volunteers were recruited from five altitudes (0, 1,600, 2,500, 3,500, and 4,500 m). All volunteers worked for 7 days at altitude, followed by 7 days of rest at sea level. We performed anthropometric assessments, nocturnal oximetry, sleep quality and sleepiness surveys, and serum melatonin levels upon awakening. Although oxygen saturation progressively decreased and heart rate increased at higher altitudes, subjective perception of sleep quality was not significantly different, and sleepiness increased in all groups compared to population at sea level. Similarly, melatonin levels increased at all assessed altitudes compared to the population at sea level. These data confirm that sleep disturbances associated with CIHH increase morning melatonin levels. Therefore, this hormone and could potentially serve as a biomarker of sleep quality.

## Introduction

Approximately 140 million people worldwide live or work at altitudes over 2,500 m, exposing them to *hypoxia*, a condition where oxygen availability is reduced due to decreased atmospheric pressure (Moore et al., 2001). According to exposure time, hypoxia can be *acute* (exposure for hours or days), *chronic* (permanent exposure to low oxygen), or intermittent (alternated periods of exposure to normoxia and hypoxia, Viscor et al., 2018). The effects of hypoxia due to altitude on the human body depend on the altitude reached, the rate of ascent, the exposure time, and the personal response to altitude (Davis and Hackett, 2017).

Mining activity is one of the most important economic incomes in Chile. Most of the mining complexes are located at altitudes between 3,000 and 5,200 m. Most workers (estimated at 120,000 people in big mining companies) have to commute rapidly (within a few hours) from lowland cities to high altitude facilities (Consejo de Competencias Mineras, 2017). Miners with this type of working shift are exposed to a model of hypoxia called *chronic intermittent hypobaric hypoxia* (CIHH), also known as *The Chilean Model* (Richalet et al., 2002). This exposure pattern is gaining attention due to the potential adverse effects of long-term exposure on cardiovascular, respiratory, and metabolic health (Brito et al., 2007; Vearrier and Greenberg, 2011; Vinnikov et al., 2011; Pedreros-Lobos et al., 2021).

Several studies show sleep disturbances in people exposed to hypoxia. Climbers exposed to acute hypoxia show sleep disturbances due to low oxygen availability from an altitude of 2,400 m, like sleep apnea episodes, or *periodic breathing* (cyclic oscillations in ventilation amplitude including apnea episodes, also known as *Cheyne-Stokes breathing*, Waggener et al., 1984). These alterations are increased at very high altitudes (West et al., 1986) and are practically absent in natives (Lahiri et al., 1983). Using simulated normobaric hypoxia, acute exposure to 4,500 m resulted in a relationship between poor sleep quality and altered mood and cognitive function (De Aquino Lemos et al., 2012).

Richalet et al. (2002) showed perceived low sleep quality in a CIHH population during the first two nights of shift work, and this perception was kept with no acclimation during the time of study (almost 2 years). Previous data from our group showed that an acclimatized population exposed to 4,200 m presented reduced arterial oxygen saturation, periodic breathing, increased awakenings, and perceived poor sleep. These alterations were improved with oxygen supplementation during the night (Moraga et al., 2014). Poor sleep quality due to nocturnal hypoxia is an important threat to lifespan, because it is an important factor related to the increased frequency of occupational accidents and its relation with an increase in risk of road traffic accident at high altitude location (Behn and De Gregorio, 2020).

Regulation of sleep is dependent on secretion of melatonin, an hormone produced from tryptophan by the pineal gland at night (Arendt et al., 1982). Production of melatonin is synchronized by the photopigment melanopsin, contained in intrinsically photosensitive retinal ganglion cells, where light exposure in the range 460–480 nm initiates the signal transduction toward several regions in the brain, including the suprachiasmatic nuclei (Do, 2019). Activation of this structure is essential to synchronize pineal secretion of melatonin and initiate the nocturnal phase of the circadian cycle (Arendt, 1998). Effects of melatonin include regulation of metabolism and reduction of energy expenditure (Coomans et al., 2013; Peschke et al., 2013; Cipolla-Neto et al., 2014), thermoregulation (Gubin et al., 2009), and redox regulation, acting as a natural antioxidant (Tan et al., 1993, 2013) and inducing expression of antioxidant enzymes (KȩKȩdziora-Kornatowska et al., 2007).

Previous studies show that hypoxia alters the circadian cycle. Acute exposure to hypoxia (2 h at 8,000 m) elevated melatonin levels in rats and resulted in remodeling of the pineal gland, which lasted between 14 and 21 days after exposure (Kaur et al., 2002). In humans, an expedition to the Himalayas shown an increase in the excretion of 6-hydroxymelatonin, a breakdown product of melatonin, indicating elevated hormone production during high altitude exposure (Frisch et al., 2004). Furthermore, patients with obstructive sleep apnea syndrome (OSAS), a clinical condition characterized by chronic and intermittent hypoxia during sleep, an elevated plasma melatonin concentration was observed, and the use of CPAP enhanced arterial oxygenation and reduced plasma melatonin concentration (Hernández et al., 2007). In this sense, acute hypoxia or high altitude exposure could affect melatonin rhythm by a delay the phase of melatonin rhythm (Coste et al., 2009) and exposure to CIHH affects circadian pattern in people exposed to 7 days of work at 4,000 m (Vargas et al., 2001). However, studies on melatonin production in miners exposed to CIHH are scarce. Therefore, the aim of this study was to evaluate melatonin levels in workers exposed to different altitudes in a model of CIHH exposure.

## Subjects, Materials, and Methods

A cross-sectional study and descriptive scope were carried out in a acclimatized workers exposed to CIHH model with a time of exposure between 6 month at 4 years. We recruited 288 male workers who worked at different altitudes (0, 1,600, 2,500, 3,500, and 4,500 m) through their respective company headquarters. All volunteers had to be working for at least 6 months at the respective altitude and included mining operations and administrative employees in diurnal work shift (starting between 7 and 8 a.m.). Shift work in this population is characterized by 7 days of work at the indicated altitude followed by 7 days of rest at lower altitude (<1,000 m). All volunteers were healthy, without diagnosis of cardiorespiratory and metabolic illness. At the time of recruitment, the volunteers read the informed consent and were informed of possible risks and discomfort, according to guidelines of the Ethics Committee of University’s Faculty of Medicine (CECFAMED# 01/09) and the Medical Director of the respective company. Once agreed, all evaluations were performed during the third day of respective shift work at high altitude as previously described (Moraga et al., 2014; Moraga et al., 2018; Pedreros-Lobos et al., 2021).

### Anthropometric Measurement

Weight (kg) and height (m) were measured using a scale (SECA model 767), and body mass index (BMI) was calculated using formula BMI = weight/height^2^ (kg/m^2^). Waist circumference (WC, cm) was measured using inextensible metric tape (SECA model 201). Four skinfolds (biceps, triceps, subscapular, and suprailiac) were determined by skinfold caliper (Lang skinfold, Cambridge, Maryland), and body fat mass (BF, percentage) was calculated using a previous described formula (Durnin and Womersley, 1973). Additionally, to estimate physical activity in the workplace, an IPAQs survey (International Physical Activity Questionary short, Ainsworth et al., 2000) was used and lifestyle was classified as sedentary, light, moderate, and active. All measurements were carried out by the same evaluator as performed previously (Pedreros-Lobos et al., 2021).

### Nocturnal Oximetry

Evaluation of nocturnal oximetry was performed using a pulse oximeter (WristOx 3100,™ Nonin, Plymouth, MN, United States). To evaluate desaturation events during the night, we considered the following variables: arterial oxygen saturation (SaO_2_, percentage), heart rate (bpm), number of events (NE), total time of events (TTE, min), average time per event (ATE, s), and oxygen desaturation index of 4% (ODI, events/h), defined by a reduction of oxygen saturation over 4% by at least 10 s.

### Sleep Quality and Sleepiness Surveys

To determine sleep quality and sleepiness, translated self-reported surveys were performed. To evaluate sleep quality, we used the Spiegel Sleep Questionnaire (Spiegel, 1984; Moraga et al., 2014), considering sleep quality as *Normal* (score below 13 points), *Regular* (between 13 and 18 points), *Poor* (between 19 and 25 points), and *Very Poor* (over 26 points). To evaluate sleepiness, we used the Epworth Sleepiness Scale (Johns, 1991), considering sleepiness as *Normal* (below 6 points), *Mild* (score between 6 and 13 points), *Moderate* (between 14 and 19 points), and *Severe* (between 20 and 24 points).

### Blood Sample and Melatonin Quantification

Blood samples were taken from the brachial vein before the work shift (between 7 and 8 am) and collected in tubes without anticoagulant. Afterward, blood samples were centrifugated (3,000 rpm by 20 min) and serum was frozen at −80°C until analysis. Melatonin levels were measured by enzyme-linked immunoassay using a commercial kit (RE54021; IBL-Hamburg GmbH, Hamburg, Germany), after treatment of samples following instructions from the manufacturer. All samples were measured in duplicate and level of melatonin was expressed in pg/mL.

### Statistical Analysis

All data were analyzed using Prism 8.4 software (GraphPad Software, San Diego, United States), tables are presented as mean ± standard deviation (SD) and plots are expressed as box-and-whisker graphs. Differences between the population at each altitude were determined using Kruskal-Wallis for non-parametric variables, followed by *post hoc* Dunn’s test. Linear regression analysis was performed to establish an association between nocturnal oximetry parameters, heart rate, sleep quality and sleepiness (as independent variables), and melatonin concentration, and correlation between parameters were performed using Pearson’s correlation coefficient. Significant differences were set at *p* < 0.05.

## Results

From 288 volunteers recruited at all altitudes, 209 were analyzed and 79 were excluded. Exclusion criteria considered people who retracted or were absent at the time of assessment, or whose nocturnal oximetry data was inconsistent due to mechanical artifacts and lipemic or hemolyzed blood samples. According with inclusion criteria, there were no records of people with chronic diseases (hypertension, diabetes, or respiratory diseases). The summary of the physical characteristics of the study population is shown in Supplementary Table 1. Briefly, mean age of all participants is 38.9 ± 9.7 years, and most of them present overweight according to BMI (27.5 ± 3.3). No significant differences in age and anthropometrical data were found between groups at altitudes studied. In agree with the IPAQs survey, 75% of volunteers were declared sedentary, 17% did light or moderate training, and only 8% were considered active.

**FIGURE 1:**
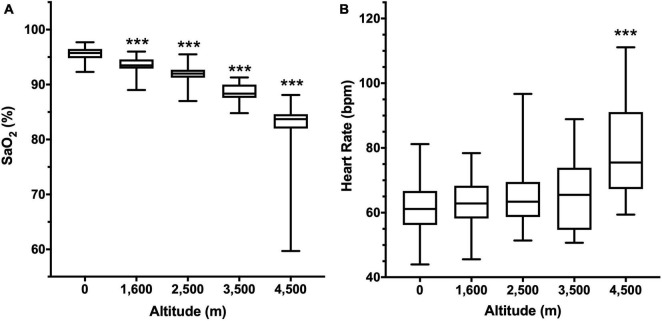
Arterial oxygen saturation (SaO_2_, **A**) and Heart rate **(B)** according to altitude. Data are presented as a box-and-whisker plot. Significant differences: ***p < 0.001 compared to the population at 0 m.

A progressive decrease in nocturnal oxygen saturation was observed, starting with 95.6 ± 1.2% in the population at 0 m, and reaching 88.5 ± 1.8% at 4,500 m (Figure 1A). Even though heart rate progressively rises with altitude, it was only significantly different at 4,500 m (78.9 ± 2.95 bpm, compared to 61.6 ± 7.8 bpm at 0 m, Figure 1B).

Nocturnal oximetry parameters trended to increase with altitude (Table 1 and Supplementary Figure 1). Desaturation events significantly rose from 2,500 m (63.4 ± 48.0), reaching a mean of 359.3 ± 262.1 events at 4,500 m (compared to 44.7 ± 66.7 at 0 m, Supplementary Figure 1A). The total time (TTE) significantly increased from 3,500 m (72.5 ± 49.6 min) and 4,500 m (113.2 ± 76.3 min) compared to 0 m (33.6 ± 45.8 min, Supplementary Figure 1B). The average time per event (ATE) was progressively reduced from 47.5 ± 20.3 s at 0 m, reaching 24.9 ± 6.2 at 3,500 m and 20.3 ± 4.1 at 4,500 m (Supplementary Figure 1C). ODI, an estimate of desaturation events greater than 4% per hour, significantly increased at 2,500 m (9.2 ± 7.0 events/h), 3,500 m (25.1 ± 19.6 events/h), and 4,500 m (46.1 ± 33.6 events/h) compared to 0 m (6.8 ± 9.7, Supplementary Figure 1D).

**TABLE 1:** Nocturnal oximetry parameters according to altitude.

Altitude (m)	N	SaO_2_ (%)	Heart rate (bpm)	NE	TTE (min)	ATE (s)	ODI (event/h)
0	60	95.6 ± 1.2	61.6 ± 7.8	44.7 ± 66.7	33.6 ± 45.8	47.5 ± 20.3	6.8 ± 9.7
1600	60	93.6 ± 1.3***	62.8 ± 7.1	61.1 ± 68.8	34.9 ± 34.2	37.7 ± 8.2	8.6 ± 9.6
2500	49	91.9 ± 1.7***	64.7 ± 9.0	63.4 ± 48.0*	32.6 ± 23.2	33.5 ± 11.4***	9.2 ± 7.0*
3500	19	88.5 ± 1.8***	66.6 ± 11.7	196.3 ± 157.3***	72.5 ± 49.6***	24.9 ± 6.2***	25.1 ± 19.6***
4500	21	82.3 ± 5.4***	78.9 ± 14.2***	359.3 ± 262.1***	113.2 ± 76.3***	20.3 ± 4.1***	46.1 ± 33.6***

Values are expressed such as mean ± SD. SaO_2_, arterial oxygen saturation; NE, number of events; TTE, total time of events; ATE, Average time per event; ODI, Oxygen desaturation index of 4%. Significant differences: *p < 0.05, ***p < 0.001 compared to the population at 0 m.

**FIGURE 2:**
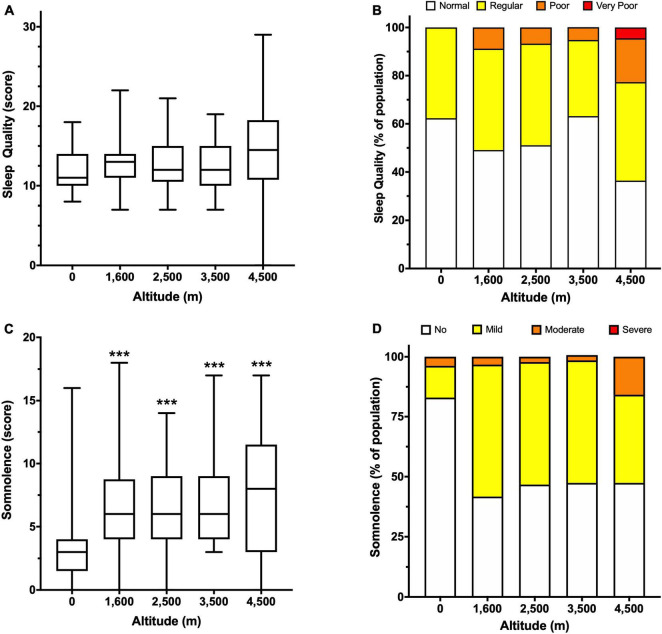
Subjective evaluation of sleep quality and somnolence according to altitude. Sleep quality is expressed as a score **(A)** and percentage of people in the indicated category **(B)**. Somnolence is expressed as a score **(C)** and a percentage of people in the indicated category **(D)**. Data in panels **(A,C)** are presented as a box-and-whisker plot. Significant differences: ***p < 0.001 compared to the population at 0 m.

Subjective sleep quality and sleepiness were analyzed using self-reported surveys at several altitudes. Although there is no significant difference in sleep quality between groups, the mean scores indicate that overall perception is “regular” (Figure 2A). However, when scores are pooled and expressed as percentages, there is a reduction in people with normal or regular sleep quality, and an increase in people with poor sleep quality at 4,500 m (Figure 2B). In addition, high altitude increased sleepiness scores, showing a significant difference at 3,500 (7.5 ± 4.1) and 4,500 m (7.8 ± 5.0) compared to 0 m (3.4 ± 3.1, Figure 2C), and although most people described sleepiness as mild, at 3,500 and 4,500 m there is an increase in the percentage of people with moderate somnolence (Figure 2D).

**FIGURE 3:**
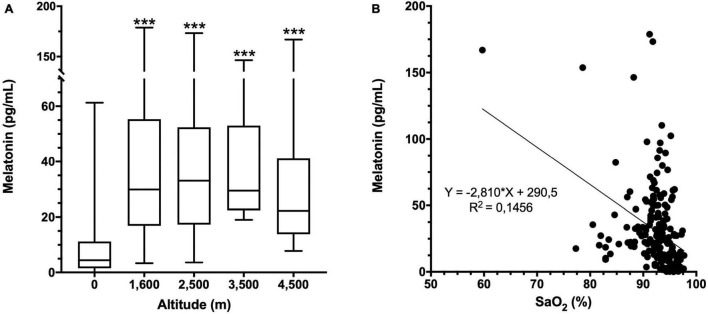
Melatonin concentration and altitude. **(A)** Melatonin concentration in plasma at each altitude. Data are presented as a box-and-whisker plot. Significant differences: ***p < 0.001 compared to the population at 0 m. **(B)** Correlation of melatonin concentration with oxygen saturation (SaO_2_) at all altitudes. Equation and the linear regression R^2^ are shown.

Melatonin concentration in miners at several altitudes was significantly increased at all altitudes compared to 0 m (8.8 ± 11.3 pg/mL). However, the mean melatonin values were similar and not significantly different between altitudes (Figure 3A and Supplementary Table 2). Compared with the parameters of nocturnal oximetry, heart rate or self-reported questionaries, we found an inverse correlation between melatonin concentration and nocturnal oxygen saturation (Figure 3B). Still, when data were separated by altitude, a significant correlation was found only at 4,500 m (Supplementary Table 2). In addition, no correlation between melatonin levels and anthropometric parameters (BMI, BF, WC) was found at any altitude studied (Supplementary Table 3).

## Discussion

This study provides new insights regarding melatonin levels in workers exposed to CIHH in Chile. The data shown here suggest that altitude could elevate melatonin levels, affecting sleep quality and increasing a risk of road traffic accidentality.

The melatonin levels observed at the time of sampling at 0 m are consistent with previous studies focused on its secretion profile, and correspond to approximately 25% of the maximum secretion level and could be used as a reference parameter (Arendt et al., 1982; Wehr, 1991; Benloucif et al., 2005). Elevation on melatonin levels at high altitude are in agree with previous reports observed in humans (Tapia et al., 2018) and rats (Kaur et al., 2002). In addition, an inverse relation was observed when arterial oxygen saturation was measured during sleep, showing that subjects that have a lower arterial oxygen saturation correlate with higher values of melatonin, especially at 4,500 m (Figure 3 and Supplementary Table 2). A similar pattern was described in a previous study were exposure to normobaric hypoxia of 13.5% oxygen (equivalent to 4,500 m) showed that increased melatonin levels are inversely correlated with arterial oxygen saturation, and exercise partially reduces this increase, improving sleep (De Aquino Lemos et al., 2018).

Evidence for the effect of altitude on sleep was well documented but focused mainly on dwellers with acute exposure to extreme altitudes, where high altitude induces sleep apneas, periodic breathing, and sleep fragmentation (reviewed in Ainslie et al., 2013). Initial studies show that people exposed to CIHH in Chile presented values of sleep quality qualified as “regular” (13–18 points) during a 7-day shift at 4,500 m, with no changes in this pattern for at least for 18 months (Richalet et al., 2002). Similar values were described in our study, supporting that sleep quality is not acclimatized. In order to enhance sleep quality at high altitude we performed a study where room enrichment with oxygen at 24% was useful to reduce apneas and improve sleep quality (with a significant reduction in mean score equivalent to “normal,” below 13 points) in a population working at 4,200 m (Moraga et al., 2014). In the present study, subjective perception of sleep was not significantly different between altitudes and did not directly correlate with arterial oxygen saturation. However, most people described regular sleep quality and mild somnolence. Possible explanations could be associated with the mining culture, where workers will not admit fatigue or sleep problems because of fears of being suspended or fired from work and a lack of objective methods to determine sleep quality. Therefore, an alternative to assess sleep quality or detect problems during sleep in the mining population is required. It is known that circadian dysregulation caused by hypoxia (Mortola, 2017) and desynchronization by shift work (Mirick et al., 2013) may represent a factor involved in the increase of risk of road traffic accidentality in high altitude activities, such as mining, border, or astronomical labor (Behn et al., 2014; Behn and De Gregorio, 2020).

Even though high altitude disturbs sleep, studies linking melatonin levels in people living or working in this condition are scarce and mostly focused on acute exposure. A simulation of 8,000 m exposure in rats showed increased melatonin production during the first 7 days, followed by a decrease to baseline levels between 14 and 21 days after exposure (Kaur et al., 2002). Another study focused on chronic and intermittent hypobaric hypoxia showed that rats exposed to 4,500 m for 32 days (in cycles of 96 h) increased melatonin levels, and this elevation was associated with antioxidant protection in the heart, lungs, and kidneys (Farías et al., 2012). However, the main source of information on melatonin production and hypoxia comes from studies related to obstructive sleep apnea syndrome (OSAS), a model of intermittent hypoxia related to upper airway obstruction. Previous studies in animals suggest that heart damage stimulated by OSAS can be prevented by treatment with melatonin (Xie et al., 2015). Additionally, the use of exogenous melatonin in rats exposed intermittently to 5% oxygen stimulates the expression of antioxidant enzymes and reduces the presence of oxidative damage markers (Hung et al., 2008). Also, the use of exogenous melatonin in rats chronically exposed to 10% hypoxia could attenuate elevated right ventricular systolic pressure, reduce pulmonary hypertension, and inhibit the expression of pro-inflammatory substances (Hung et al., 2017). Preliminary studies show decreased expression of pro-inflammatory cytokines interleukin 2 (IL-2) and IL-6 measured in lymphocytes of rats exposed to CIHH of 4600 m, which could be associated with increased anti-inflammatory interleukin-10 levels (Bernal et al., 2009). In regard to sleep biomarkers and its regulation, supporting evidence shows that increased levels of IL-1 and tumor necrosis factor alfa (TNF-α) can modulate a complex biochemical cascade that regulates sleep in humans and animals, and IL10 exerts an inhibitory effect of over sleep control (to more details, see Clinton et al., 2011). Related with this, unpublished data from our group suggest increased anti-inflammatory interleukin-10 production in people with exposure to CIHH at 4,500 m. This cytokine could be part of a protective response to high altitude-mediated inflammation and oxidative damage, but more studies are needed to confirm the role of melatonin. One hypothesis could be related to alterations in sympathetic pathways promoted by hypoxia since melatonin synthesis is stimulated by norepinephrine in the absence of light (Arendt, 1998). Stressful stimuli, such as hypoxia itself or in combination with exercise, can stimulate interleukin-6 expression, mediated by the α-adrenergic pathway (Mazzeo et al., 2001). Furthermore, melatonin production can be peripherally enhanced in dendritic cells and macrophages by activating β-adrenoreceptors, turning them to an anti-inflammatory state (Pires-Lapa et al., 2018), supporting the idea of an interaction between hypoxia, the immune system, and melatonin production.

Another important factor associated with melatonin production is light exposure, which inhibits production and delays sleep induction (Lewy et al., 1980). Exposure to light at a wavelength between 446 and 477 nm (near blue light in the spectrum, as seen in electronic devices such as tablets or smartphones) suppresses melatonin production during the dark phase of the circadian cycle (Brainard et al., 2001). This phenomenon was evaluated as a potential regulator of the cycle, helping acclimatization during acute exposure to hypoxia (Silva-Urra et al., 2015). In this work, exposure to blue light during simulated hypoxia equivalent to 4,000 m increased systolic blood pressure and improved cognitive performance and alertness, associated with inhibited melatonin production. Another factor to consider is the time of light exposure, mainly associated with seasonal variation. Exposure to light is necessary to synchronize the cycle, affecting the duration of wakefulness, shortening the duration of sleep time in summer and extending it in winter. This seasonal pattern shifts melatonin production without modifying the amount of plasma melatonin (Wehr, 1991). Since all volunteers performed daytime work during the study and were exposed to similar artificial light in the common areas inside the complexes, most participants probably exhibited a similar photoperiod. Therefore, it would not be a relevant factor affecting hormone production, but personal habits in their bedrooms, including the use of television or mobile devices before sleeping, could affect melatonin production (Oh et al., 2015; Yasukouchi et al., 2019).

The limitations of the present study are associated with the melatonin production profile in miners during shift work. To precisely establish whether alterations in melatonin are associated with changes in the nocturnal phase, 24 h studies are necessary. However, taking blood samples during the shift is difficult, and we could not evaluate the nocturnal secretion profile or perform multiple blood sampling due to technical limitations and restrictions imposed by companies and labor unions. Although saliva samples are considered a good replacement for blood as a source of melatonin, daytime production could be too low to detect a significant amount unless alterations in secretion increase availability.

In conclusion, evaluation of melatonin levels in wakefulness could be used as a marker to analyze sleep quality at high altitude and a possible evidence of protection against oxidative damage in workers exposed to CIHH. Future studies should consider the long-term effect of CIHH exposure on melatonin levels and assess the potential prophylactic role of melatonin supplementation as an element that improves sleep quality and function as a cardiac and pulmonary protector associated to high altitude exposure.

## Data Availability Statement

The original contributions presented in the study are included in the article/Supplementary Material, further inquiries can be directed to the corresponding author.

## Ethics Statement

The studies involving human participants were reviewed and approved by Ethics Committee of the Facultad de Medicina, Universidad Católica del Norte, Chile. The participants provided their written informed consent to participate in this study.

## Author Contributions

RC-J and FM conceived and designed the study, and performed the statistical analysis. DM and RC-J contributed to sample and data collections. FM was the guarantor. All authors drafted the report, interpreted the results, critically revised the manuscript, and approved the final manuscript.

## Conflict of Interest

The authors declare that the research was conducted in the absence of any commercial or financial relationships that could be construed as a potential conflict of interest.

## Publisher’s Note

All claims expressed in this article are solely those of the authors and do not necessarily represent those of their affiliated organizations, or those of the publisher, the editors and the reviewers. Any product that may be evaluated in this article, or claim that may be made by its manufacturer, is not guaranteed or endorsed by the publisher.
