# Supplementary material for: The Effect of Chronic Intermittent Hypobaric Hypoxia on Sleep Quality and Melatonin Serum Levels in Chilean Miners
Source: Front Physiol. 2022 Feb 9;12:809360. doi: 10.3389/fphys.2021.809360 (PMC8864145; doi:10.3389/fphys.2021.809360)

Supplementary Figure 1: Nocturnal oximetry parameters according to altitude. (A) TE, number of events. (B) TTE, total time of events. (C) ATE, Average time per event. (D) ODI, Oxygen desaturation index of 4%. Data are presented as a box-and-whisker plot. Significant differences: * p<0.05, ** p<0.01, *** p<0.001 compared to the population at 0 m.

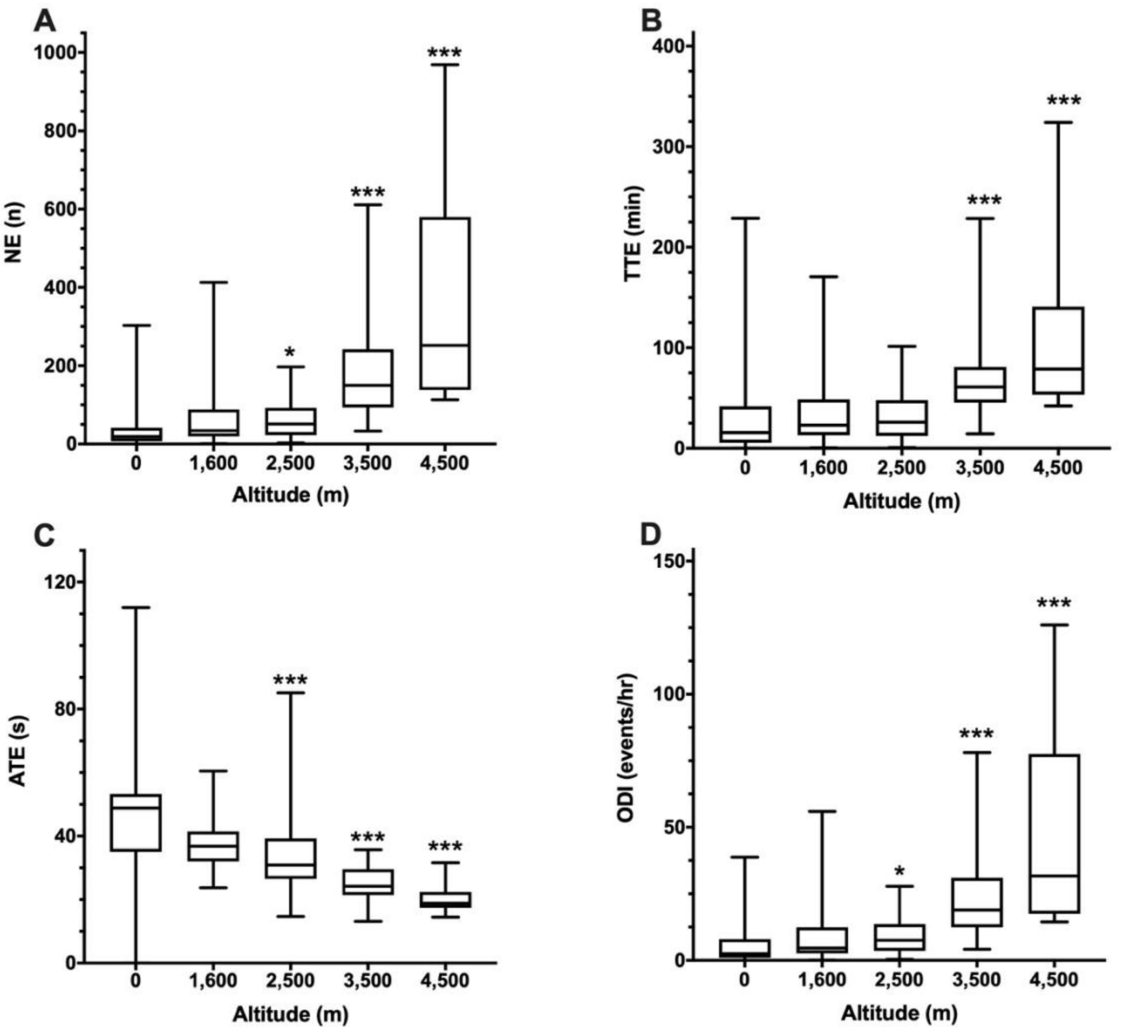

Supplement: Supplementary file 1 [file Image_1.pdf]
