# Supplementary material for: The Effect of Chronic Intermittent Hypobaric Hypoxia on Sleep Quality and Melatonin Serum Levels in Chilean Miners
Source: Front Physiol. 2022 Feb 9;12:809360. doi: 10.3389/fphys.2021.809360 (PMC8864145; doi:10.3389/fphys.2021.809360)
Supplement: Supplementary file 2 [file Table_1.docx]

Supplementary Table 1. Anthropometric measurements of volunteers.

| Altitude (m) | N | Age  (years) | Weight  (kg) | Height  (m) | BMI  (kg/m^2^) | WC  (cm) | BF  (%) |
| --- | --- | --- | --- | --- | --- | --- | --- |
| ALL | 209 | 38.9 ± 9.7 | 81.0 ± 11.9 | 1.72 ± 0.06 | 27.5 ± 3.3 | 95.2 ± 9.2 | 27.2 ± 4.8 |
| 0 | 60 | 42.2 ± 10.7 | 80.0 ± 13.2 | 1.71 ± 0.07 | 27.2 ± 3.7 | 95.5 ± 9.6 | 26.8 ± 5.0 |
| 1600 | 60 | 37.0 ± 9.1 | 82.7 ± 12.0 | 1.73 ± 0.06 | 27.7 ± 3.1 | 95.8 ± 9.6 | 27.4 ± 4.4 |
| 2500 | 49 | 36.6 ± 9.1 | 79.5 ± 11.8 | 1.69 ± 0.06 | 27.6 ± 3.5 | 93.5 ± 9.2 | 26.3 ± 5.2 |
| 3500 | 19 | 41.2 ± 10.0 | 78.2 ± 8.7 | 1.71 ± 0.03 | 26.9 ± 3.1 | 95.2 ± 7.2 | 30.2 ± 4.5 |
| 4500 | 21 | 38.2 ± 8.6 | 84.7 ± 9.6 | 1.75 ± 0.05 | 27.5 ± 2.7 | 98.1 ± 8.7 | 27.5 ± 2.9 |

Data are presented as Mean ± SD. BMI, Body Index Mass; WC, Waist circumference; BF, Body fat mass.
