# Supplementary material for: The Effect of Chronic Intermittent Hypobaric Hypoxia on Sleep Quality and Melatonin Serum Levels in Chilean Miners
Source: Front Physiol. 2022 Feb 9;12:809360. doi: 10.3389/fphys.2021.809360 (PMC8864145; doi:10.3389/fphys.2021.809360)
Supplement: Supplementary file 3 [file Table_2.docx]

Supplementary Table 2: Lineal regression analysis and Pearson’s correlation between melatonin concentration and arterial oxygen saturation (SaO_2_).

| Altitude (m) | N | Slope | Y-intercept | X-intercept | R^2^ | r | P value |  |
| --- | --- | --- | --- | --- | --- | --- | --- | --- |
| ALL | 209 | -2.81 | 290.5 | 103.4 | 0.1456 | -0.467 | <0,001 | *** |
| 0 | 60 | -0.40 | 47.1 | 117.6 | 0.0018 | -0.042 | 0.75 |  |
| 1600 | 60 | -4.51 | 462.6 | 102.5 | 0.0344 | -0.185 | 0.16 |  |
| 2500 | 49 | -0.19 | 54.0 | 288.3 | 0.0001 | -0.011 | 0.94 |  |
| 3500 | 19 | -0.97 | 129.7 | 133.1 | 0.0025 | -0.050 | 0.84 |  |
| 4500 | 21 | -6.21 | 542.3 | 87.3 | 0.5862 | -0.766 | 0.004 | ** |

Significant differences of Pearson’s correlation: * p<0.05, ** p<0.01, *** p<0.001.
