# Supplementary material for: The Effect of Chronic Intermittent Hypobaric Hypoxia on Sleep Quality and Melatonin Serum Levels in Chilean Miners
Source: Front Physiol. 2022 Feb 9;12:809360. doi: 10.3389/fphys.2021.809360 (PMC8864145; doi:10.3389/fphys.2021.809360)
Supplement: Supplementary file 4 [file Table_3.docx]

Supplementary Table 3: Lineal regression analysis and Pearson’s correlation for melatonin concentration and anthropometric parameters.

| BMI and Melatonin | | | | | | | | | | | | | | | | |
| --- | --- | --- | --- | --- | --- | --- | --- | --- | --- | --- | --- | --- | --- | --- | --- | --- |
| Altitude (m) | N | | Slope | | Y-intercept | | X-intercept | | R^2^ | | r | | P value | |  | |
| ALL | 209 | | -0.16 | | 34.3 | | 215.8 | | 0.0003 | | -0.017 | | 0.81 | |  | |
| 0 | 60 | | 0.23 | | 2.55 | | -11.11 | | 0.0058 | | 0.076 | | 0.56 | |  | |
| 1600 | 60 | | 1.04 | | 11.37 | | -10.88 | | 0.0099 | | 0.099 | | 0.45 | |  | |
| 2500 | 49 | | -2.19 | | 97.94 | | 44.66 | | 0.0760 | | -0.276 | | 0.06 | |  | |
| 3500 | 19 | | -0.72 | | 63.06 | | 87.80 | | 0.0043 | | -0.066 | | 0.79 | |  | |
| 4500 | 21 | | -2.00 | | 89.12 | | 44.53 | | 0.0206 | | -0.143 | | 0.61 | |  | |
| BF and Melatonin | | | | | | | | | | | | | | | | |
| Altitude (m) | N | | Slope | | Y-intercept | | X-intercept | | R^2^ | | r | | P value | |  | |
| ALL | 209 | | -0.03 | | 30.99 | | 1090.00 | | 1.96e-05 | | -0.004 | | 0.95 | |  | |
| 0 | 60 | | 0.01 | | 8.44 | | -618.00 | | 3.61e-05 | | 0.006 | | 0.96 | |  | |
| 1600 | 60 | | 0.56 | | 25.22 | | -44.67 | | 0.0058 | | 0.076 | | 0.56 | |  | |
| 2500 | 49 | | -1.77 | | 83.95 | | 47.39 | | 0.1111 | | -0.333 | | 0.02 | | * | |
| 3500 | 19 | | 0.29 | | 34.29 | | -116.30 | | 0.0016 | | 0.039 | | 0.87 | |  | |
| 4500 | 21 | | 3.93 | | -69.70 | | 17.73 | | 0.0386 | | 0.194 | | 0.59 | |  | |
| WC and Melatonin | | | | | | | | | | | | | | | | |
| Altitude (m) | | N | | Slope | | Y-intercept | | X-intercept | | R^2^ | | r | | P value | |  |
| ALL | | 209 | | -0.06 | | 36.83 | | 612.4 | | 0.0003 | | -0.018 | | 0.80 | |  |
| 0 | | 60 | | 0.07 | | 2.11 | | -28.51 | | 0.0035 | | 0.059 | | 0.68 | |  |
| 1600 | | 60 | | 0.41 | | 0.94 | | -2.32 | | 0.0145 | | 0.120 | | 0.36 | |  |
| 2500 | | 49 | | -0.75 | | 106.70 | | 142.70 | | 0.0636 | | -0.252 | | 0.08 | |  |
| 3500 | | 19 | | -0.57 | | 97.26 | | 171.30 | | 0.0150 | | -0.122 | | 0.62 | |  |
| 4500 | | 21 | | -0.005 | | 40.50 | | 8301.0 | | 9,15e-07 | | -0.001 | | 0.99 | |  |

Significant differences of Pearson’s correlation: * p<0.05.
